# A qualitative study of bereaved parents and healthcare professionals on perinatal loss

**DOI:** 10.18332/ejm/194159

**Published:** 2024-12-19

**Authors:** Jozefiene Jansens, Kristof Faes, Maëlle De Coninck, Joni Gilissen, Liesbeth Van kelst

**Affiliations:** 1 Research Centre Care in Connection, Department of Nursing and Midwifery, Karel de Grote University of Applied Sciences and Arts, Antwerp, Belgium; 2 Equity in Brain Health, Atlantic Institute, Oxford, United Kingdom; 3 Department of Family Medicine and Chronic Care, Vrije Universiteit Brussel, Brussels, Belgium; 4 Department of Public Health and Primary Care, Universiteit Ghent, Ghent, Belgium

**Keywords:** perinatal loss, perinatal care, midwifery, mental health, bereavement, palliative care

## Abstract

**INTRODUCTION:**

Perinatal loss, encompassing stillbirth and neonatal death, can have profound physical and psychological consequences for parents. Effective communication by healthcare professionals during this sensitive period is critical. This study aimed to explore how bereaved parents and professionals experienced verbal and non-verbal communication during perinatal loss.

**METHODS:**

A qualitative, in-depth interview study following grounded theory principles was conducted in Flanders, Belgium, between January and June 2021. Participants were purposively selected via a hospital ward. Face-to-face (n=8) and online (n=13) interviews were carried out by two midwife researchers who were aware of potential biases related to personal/professional interests. A group of bereaved parents and professionals provided feedback during the project. Qualitative analysis was conducted using NVIVO, employing open and axial coding to identify themes.

**RESULTS:**

Eleven bereaved parents and ten professionals participated. Six themes emerged: 1) navigating time and adjusting pace; 2) recognition of parenthood in woman and partner; 3) clear, honest information for shared decision-making; 4) authentic contact while leaving room for alone time; 5) gentle and sensitive verbal communication; and 6) professionals’ self-care. Parents valued clear, honest communication, acknowledgment of their parenthood, and the ability to make informed decisions. Healthcare professionals emphasized the challenge of providing adequate time and presence amidst busy schedules, but recognized the importance of empathy and sensitivity.

**CONCLUSIONS:**

Compassionate, patient-centered care with effective verbal and non-verbal communication is vital in supporting bereaved parents during perinatal loss, and it continues to be a challenge. Findings can guide clinical practice to inform professional training initiatives and inform intervention development.

## INTRODUCTION

Losing a child during pregnancy, or shortly after birth, is a devastating experience for parents and has been observed as a type of grief that is complex and often traumatic^1^. Globally, an estimated 14 stillbirths per 1000 births, and 17 neonatal deaths per 1000 births, occur^2^. In addition, early loss of pregnancy (or miscarriage) before viability is estimated at 23 million miscarriages every year, translating into 44 pregnancy losses each minute^3^.

The physical and psychological consequences of perinatal loss, in all of its forms (miscarriage, stillbirth, and neonatal death as defined below in ‘Study setting and research participants’) are well documented, with studies reporting mental health challenges^4^, higher anxiety and depression levels^4^, post-traumatic stress^5^, unresolved grief^6^, or fear of recurrence of loss^7^. Most research on the psychological impact focuses on women^8^, while there is a high need for validating perinatal grief in partners in order to provide effective emotional support for both parents^9^.

Affected women and their partners report incorrect approaches to their bereavement as an important contributing factor to mental health issues^7^. In addition to grappling with the grief of their loss, parents navigating such tragic circumstances also encounter a range of challenges in their interactions with healthcare professionals during this delicate period^10^. In particular, verbal and non-verbal communication from healthcare professionals plays a crucial role in shaping the experiences of bereaved parents. Effective communication has been identified as a key component of quality healthcare delivery^11^, potentially enhancing patient satisfaction, understanding, and coping mechanisms, particularly in sensitive and difficult situations such as perinatal loss^12^. Despite the recognized importance of accurate verbal and non-verbal communication in the care of bereaved parents, research examining their experiences of verbal and non-verbal communication from healthcare professionals during the perinatal period remains limited. Existing studies have highlighted various aspects of communication that impact parental experiences, including the delivery of sensitive information, empathy, support, and the provision of practical guidance^11,13-15^. Since studies show that parents’ experiences depend heavily on their interactions with staff^16^, we aimed to better understand what in the communication processes can be improved or is particularly hard, from the perspective of both bereaved parents and the staff confronted with these cases.

The primary aim of this study was to examine the influence of healthcare professionals’ verbal and non-verbal communication on the experiences of bereaved parents during perinatal loss, as well as to explore the experiences of the healthcare professionals themselves in these interactions.

## METHODS

### Research team and reflexivity

The research team comprised multidisciplinary professionals with diverse backgrounds. The interviews were conducted by LVK and JJ. The researchers had prior experience in qualitative research, and were experienced midwives who were trained in sensitive communication, particularly in perinatal loss. The relationship between the researchers and participants was carefully managed to minimize bias. There was no prior relationship between the interviewers and the participants. Participants were informed about the researcher’s professional background, the study’s objectives, and the purpose of the research. The researchers remained aware of potential biases and assumptions related to personal interests in perinatal bereavement care, regularly reflecting on these to maintain objectivity throughout the study. The steering committee of bereaved parents also provided feedback, ensuring that communication was handled with sensitivity.

### Study design and setting

This study was designed as a qualitative, in-depth interview study using a grounded theory approach. This approach is particularly useful in this study, as it aims to generate new insights into the interaction and communication between parents and professionals about perinatal loss. Both the Qualitative Research Review Guidelines (RATS) and COREQ criteria were followed for reporting. The research was conducted in Flanders, Belgium, between January 2021 and June 2021. According to the Study Center for Perinatal Epidemiology (SPE), the perinatal mortality rate in Flanders is 448 babies per year. Still, the actual number is higher as only births ≥500 g are registered. Data were collected from bereaved parents and healthcare professionals at various locations, including a walk-in center for those dealing with child loss, the workplaces of healthcare professionals, and participants’ homes (n=8). Online interviews were also conducted (n=13) to accommodate participants’ preferences and COVID-19 guidelines. No Non-participants were not present during the interviews to ensure confidentiality and encourage open communication. The environment was designed to be supportive and comfortable, particularly for the bereaved parents.

### Participants

The study included a purposive sample of 11 bereaved parents and 10 healthcare professionals. Eligible parents were those who had experienced the death of a child during the perinatal period within the last five years in a hospital setting (delivery room, maternity ward, neonatology, or pediatrics). The inclusion of this timeframe accounted for recent changes in perinatal loss protocols and the emergence of grassroots initiatives. Parents whose babies died outside of the hospital were excluded. It is important to note that the study did not include participants who experienced perinatal loss during the COVID-19 pandemic, recognizing that communication dynamics may differ in that context. Healthcare professionals, including midwives, nurses, gynecologists, pediatricians, and neonatologists working in hospital settings, were eligible to participate regardless of their experience with perinatal loss. The study aimed for maximum diversity by selecting participants based on criteria such as the timing of death (during pregnancy, childbirth, or post-childbirth), the department, and the professional role of healthcare providers.

Participants were approached through written invitations distributed via grassroots initiatives for parents and professional umbrella associations for healthcare providers, including the Flemish Professional Organization for Midwives (VBOV), Flemish Intensive Neonatal Care (VINZ), and the Flemish Association for Obstetrics and Gynecology (VVOG). The recruitment process involved direct contact with researchers after receiving the invitation, followed by providing detailed study information and collecting informed consent. Participants were selected until data saturation was reached, with at least ten parents and ten healthcare professionals participating.

### Data collection

Data were collected through in-depth, semi-structured interviews with parents and healthcare professionals, guided by a pre-structured topic list. The guide included open-ended questions to explore experiences and perceptions related to professional–parent communication during perinatal loss. Specific attention was given to key moments, such as the announcement of the child’s passing or the first interaction with the deceased child. Questions addressed verbal and non-verbal communication, prompting participants to share their feelings, perceptions, and reflections. The guide was developed through a collaborative process involving a pilot interview with experts from the resonance group and parents from the steering committee to ensure relevance and sensitivity to the topic.

For healthcare professionals, the interview guide focused on their experiences and perceptions when communicating with bereaved parents and their needs and challenges in providing care during these sensitive situations.

All interviews were audio-recorded with the participants’ consent, and field notes were taken during and after each interview to capture non-verbal cues and contextual details. The duration of each interview varied, ranging from 26 to 108 minutes. Data saturation was achieved after 21 interviews, and no repeat interviews were necessary.

### Data analysis

The data analysis followed grounded theory principles, utilizing NVIVO 13 software for inductive analysis. The research team, consisting of two coders, conducted open coding of the transcripts, followed by axial coding to identify relationships and patterns within the data. A coding tree was developed and iteratively refined throughout the analysis process. Member checks were conducted by sharing preliminary findings with the resonance group of healthcare professionals, who provided feedback to ensure the findings resonated with their experiences. This process helped to validate the themes and enhance the credibility of the results.

### Definitions

In this study, the term ‘perinatal loss’ was used to encompass all instances of prenatal and early neonatal mortality. ‘Stillbirth’ was defined as the spontaneous intrauterine death of a fetus weighing ≥500 g and/or occurring after 16 weeks of pregnancy. ‘Early neonatal mortality’ referred to the death of a live-born child weighing ≥500 g before the 8th day after birth. ‘Perinatal loss’ was defined as the combination of stillbirth and early neonatal mortality. These definitions align with established medical terminology and are referenced accordingly.

### Ethics

Ethical approval for this study was obtained from the Social Sciences and Humanities Ethics Committee on 20 April 2021 (reference: SHW_20_131). Participation was voluntary, with written informed consent obtained from all participants after they were informed about the study’s purpose, procedures, risks, and benefits, via email. Privacy and confidentiality were maintained by assigning pseudonyms to all participants and anonymizing the data during analysis and reporting. Special care was taken to handle sensitive emotional content, with ongoing psychological support provided to the research team.

## RESULTS

### Participant characteristics

Data were collected from March to June 2021. The interviews included 11 bereaved parents and 10 healthcare professionals in mother–child care. Demographic and professional characteristics are shown in Table 1. The mean age of bereaved parents was 32.3 years. Nine parents experienced a loss during pregnancy, and two parents experienced early neonatal loss. Healthcare providers were 60% midwives, and the average professional experience was 14.6 years, ranging from 1 to 34 years of experience.

**Table 1:** Characteristics of participant bereaved parents involved in the in-depth interviews on perinatal loss conducted in Flanders, Belgium, 2021 (N=11)

*Characteristics*	*n*	*%*
**Gender**		
Female	10	91
Male	1	9
**Age** (years), mean (range)	32.3 (27–37)	
**Time of loss of baby**		
**During pregnancy**	9	82
Mors *in utero* (MIU)*	3	27
Termination of pregnancy (TOP)**	6	55
**Within the first 8 days after birth**	2	18

* Spontaneous intrauterine death of a fetus at any point after 16 weeks of pregnancy.

** Medical or surgical termination of a pregnancy.

**Table 2:** Characteristics of participant healthcare professionals involved in the in-depth interviews on perinatal loss conducted in Flanders, Belgium, 2021 (N=10)

*Characteristics*	*n*	*%*
**Gender**		
Female	10	100
Male	0	0
**Healthcare profession**		
Midwife	6	60
Neonatal nurse	2	20
Gynecologist	1	10
Neonatologist	1	10
**Level of experience** (years), mean (range)	14.6 (1–34)	

### Supportive bereavement care in perinatal loss

We identified six themes. Quotes that most accurately highlight the theme were selected. An effort was made to ensure diversity among the respondents when selecting these quotes.

*Theme 1: Navigating time and following the pace of parents*

All participants emphasized the importance of professionals matching the pace set by the parents when providing care. Parents expressed the desire for time that seemed to pass quickly during moments of uncertainty, yet slowly once a negative diagnosis was made, or the loss of their baby was confirmed. For every parent, the narrative of their baby’s birth commenced amidst significant uncertainty, either about the diagnosis or the status of the baby’s vitality. Parents in this study universally stipulated they wanted this uncertain period to pass by as quickly as possible. The majority expressed the need for professionals to prioritize getting news to these parents and minimize wait times as much as possible or provide support during that time:

*‘We had an appointment at nine, and we waited for two and a half hours. That is really annoying.’ (Mother 5, MIU)*

*‘We had a conversation of one and a half minute and the gynecologist said “you have to come back for an amniocentesis in two weeks, sooner is not possible ... It felt like “its’s Down’s syndrome, and now you have to wait two weeks”. There was no follow-up in those weeks.’* (Mother 6, TOP)

Once parents had certainty about the diagnosis or death, they emphasized the importance of not feeling rushed and proceeding at their own pace. Parents who opted for a termination of pregnancy (TOP) valued having ample time to absorb the situation and carefully weigh their options without time pressure. They also appreciated deciding the timing of the necessary delivery. For example, one couple went on a holiday after deciding on a TOP, feeling it was their last chance for a holiday with their baby. This need for time was also important for parents experiencing a stillbirth:

*‘Nobody rushed us or said “look, you have to decide now”. Absolutely not, on the contrary.’* (Mother 5, TOP)

*‘The gynecologist asked, “Would you like to be admitted to the hospital tonight?”. I didn’t know what to choose at the moment, but I’m glad I got more time to prepare myself.*

*I wanted to enjoy every second with her. If I had been induced that night, I would have been too overwhelmed to remember half of it now.’* (Mother 7, MIU)

*‘We could have the baby delivered two days later, but we chose to wait a week’* (Mother 11, TOP)

Parents who lost their baby in the first week of life in the NICU (n=2) had similar needs for pacing. They valued choosing how much time to spend with their baby after death and appreciated being consulted about the timing of their last moments with their baby. The parents could also determine the final moment of handing over the baby after seeing them for the last time, which was extremely important for them:

*‘She (midwife) said: “what we will do is that, when it is okay for you both, we will put her on your chest, on one of you, or both, you can switch as often as you want. And when it’s ok, we will gradually stop treatment, but totally at your pace”’* (Mother 4, ND).

*‘At one point, I think I asked for him to be brought back and forth four times in an hour, and it was always done with a smile. It was always without any issues.’* (Mother 6, TOP)

Healthcare professionals expressed a strong desire and made strong efforts to allocate ample time to support bereaved parents throughout their care trajectory, even though they acknowledged the difficulty of finding enough time amidst their busy work schedules in the delivery ward. Most stipulated the importance of being ‘present’ at the moment and conveying to parents that they were genuinely listened to and cared for:

*‘I always try to sit down and maintain a calm presence with them in that room, even if there is chaos elsewhere on the ward and everything is exploding outside, so to speak. I don’t want people to notice.’* (Professional 10, midwife)

*‘Sometimes we only need to be inside for five or ten minutes, really present in the moment. … I think in rooms where people are going through something that is so against nature ... you have to be present there too, of course. I believe it’s important to leave your phone outside for a moment. Or finish everything first and then focus on the situation there ... Because, for them the world stands still for a moment.’* (Professional 7, gynecologist)

*Theme 2: Recognition of parenthood of both woman and partner*

Despite not having a living baby, parents strongly felt the profound sense of becoming parents. As new parents, they believed congratulations were appropriate, although not universally received. Healthcare providers also recognized the importance of acknowledging new parenthood, though explicitly saying ‘congratulations’ felt unsuitable for some, presenting a challenge. This sensitivity varies depending on circumstances (e.g. miscarriage vs termination of pregnancy) and should be approached on a case-by-case basis. For instance, one midwife refrained from congratulating parents after a termination of pregnancy as they had made that decision themselves. Most healthcare providers suggested alternative ways to validate parents, such as appreciating the child’s beauty, acknowledging their efforts, or expressing pride in their journey:

*‘I remember not receiving congratulations because I vividly recall our photographer entering the room and being the first to say, “Congratulations, Mom”. I became a mom*

*… It’s a child we didn’t bring home, but it’s still a child.’* (Mother 6, TOP)

*‘It varies. Personally, I find saying ‘congratulations’ quite challenging. Instead, I often say something like, “Wow, your baby is really beautiful. Look at how he or she looks”.’ I find that approach more comfortable. Knowing when to say ‘congratulations’ and when not to is very situational and not always clear to me.’* (Professional 1, midwife)

Nearly all parents noted that healthcare professionals consistently referred to the baby by its name, a gesture greatly appreciated. One mother felt the absence of this acknowledgment when the child’s name was left unspoken. All professionals indicated they made efforts to use the baby’s name and addressed the parents as ‘mother’ and ‘father’, as they would with a living child, regardless of the pregnancy term. They also found it important to treat the baby with the same care as a living infant, including handling them gently, speaking softly, and complimenting them. Other examples mentioned included covering the baby when cold, bathing, drying, and noting the time of birth:

*‘They asked how we wanted to name him, and then used his name. They commented on his beautiful feet and similar details. Their ease with everything eased my tension. They held him first, wrapped him in a blanket, and gently handed him to me, all with loving care. It made me less afraid.’* (Mother 10, TOP)

*‘You handle them with just as much care, placing them in exactly the same spot as the parents agree. I cover them just as well, in case they get cold, and I also inform about the time. … When I check on the mother, I place the baby with the father. So, in that regard, there’s actually very little difference in what I would normally do.’* (Professional 5, midwife)

*‘I felt it was crucial for him to be acknowledged as a real presence, not in the sense of ‘he’s not alive and breathing, so he’s less valuable’. There were times when I felt more special or significant than a mother leaving with her healthy baby in a car seat.’* (Mother 6, TOP)

Two mothers felt that their partners were not adequately involved. Communication in these cases mainly centered around the mother, leaving the other parent feeling excluded. Four healthcare professionals indicated their active attempt to engage and involve the partner, ensuring equal levels of empathy or physical contact, regardless of whether they were the ones who delivered or carried the baby. One parent expressed that an additional effort towards the partner might be needed to involve them fully:

*‘… I might also be the more talkative one of us two, but it was nice that she asked him questions directly like that.’* (Mother 10, TOP)

*Theme 3: Clear and honest information to inform shared decision-making regarding diagnosis, treatment, time, and mode of birth*

Participants unanimously emphasized empowering parents to make autonomous decisions as much as possible. They stressed the need for parents to be involved in decisions regarding their care and that of their baby, including methods of pregnancy termination, birthing options, viewing and holding the baby, and arranging post-birth rituals. They highlighted the importance of receiving ample information, suggesting that no amount of information is excessive and that repetitive information is valued. Parents anticipate thorough discussions of all available options to facilitate informed decision-making. Parents expect the information provided to meet specific criteria: ‘Clarity, conciseness, and comprehensiveness’; ‘Communication in plain language, free from excessive technical jargon’; ‘Factual accuracy without euphemisms’; ‘Coverage of past, present, and future aspects of the situation’; and ‘Complete honesty from professionals, especially avoiding false assurances when uncertain about outcomes’.

Healthcare professionals corroborate the need for repeated information, noting that crucial information is lost during pivotal moments. They particularly emphasize the importance of information regarding what will happen. Most of them (especially midwives) indicated they used a bereavement toolbox developed by a grassroots initiative in Flanders, entailing tools and information for parents:

*‘In all aspects of midwifery, it’s really about listening to what the people want, but here, it’s even more crucial.’* (Professional 9, midwife)

*‘The midwife was fantastic. She left all the decisions to us, making everything available. She mentioned the bereavement suitcase from Berrefonds [community organization focused on perinatal loss], saying, “You can open it or not, it’s up to you”. She also respected our choice on having visitors. She emphasized that there’s no right or wrong in what we were doing that day.’* (Mother 9, MIU)

*Theme 4: Authentic contact with a designated healthcare professional while leaving room for alone time*

Although most parents appreciated a professional who dedicated ample time in the room with them, they experienced a need for moments alone to process the news. Consensus among parents was to minimize interactions with multiple parties, thus ensuring continuity of care. The latter was also shown in two cases where colleagues were not informed of the child’s passing, leading to inappropriate statements: a pediatrician entering a room with the message to examine the child or a colleague wishing parents success with the baby at home upon discharge:

*‘In the morning, I think, about seven people walked in, and I had slept very little the night before. Yeah, that’s really difficult. You’re completely exhausted, and you appreciate that people come to visit, but on the other hand, you just want to say, “Guys, close that door and let us take a moment to reflect on what we’re doing”.’* (Mother 6, TOP)

Agreeing that these events are emotionally challenging, most healthcare professionals acknowledged that they sometimes felt emotional and felt it appropriate not to hide it as long as they could continue to perform their duties professionally. Parents indicated that this was acceptable, sometimes even valued. Next to showing emotions, participants accepted and appreciated contact through small comforting gestures such as a hand on the shoulder or arm. Healthcare professionals viewed them as a means of building connections and ‘being there’:

*‘You have to try to hold yourself back a bit, but if you have a lump in your throat or a tear on your cheek ... I don’t think that’s unprofessional. People should be able to see that it affects you too.’* (Professional 4, neonatal nurse)

*‘At a certain moment, the midwife was also crying ... for me, it was actually very comforting.’* (Mother 5, MIU)

*‘Crying uncontrollably or loudly sobbing, I wouldn’t have appreciated that either, but shedding a tear or showing clear emotion in the eyes, that wouldn’t have bothered me.’* (Mother 7, MIU)

*‘I vividly remember my gynecologist gently rubbing my arm. It made me feel deeply supported and cared for.’* (Mother 1, TOP)

*Theme 5: Gentle and sensitive verbal communication*

All participants emphasized the importance of professionals being kind, gentle, and empathetic towards the parents (non)verbally. Based on the statements, we observed difficulties in professionals communicating not only about the death but also in supporting the parents, showing flags in physician–patient communication.

Overall, all participants found communication difficult but important for the grief process, and it was challenging. Various statements showed that the professional communication approach, despite having been identified as supportive, was often inadequate, including statements that minimized feelings, pretending as if nothing had happened, and reinforcing the idea that death is a social taboo:

*‘… all that sugarcoating, I don’t need that. Just tell it like it is, but with empathy.’* (Mother 5, MIU)

Several participants considered adjusting the tone of voice by speaking somewhat slower and softer a best practice. However, healthcare professionals did mention that it depended on the parents, and they adjusted their type of communication depending on the individual.

Both parents and healthcare professionals mentioned that professionals must be experienced or trained to provide compassionate care and use appropriate wordings while always introducing themselves to the bereaved couple. The interviewed gynecologist also mentioned that a case involving parents losing their child is better not delegated to a trainee doctor. She pointed out that during her training, she found it very difficult to discuss decisions or guide the delivery with an unfamiliar couple.

*Theme 6: Self-care of healthcare professionals*

Healthcare professionals who were confronted with the challenging aspects and pressures of caring for parents experiencing the loss of a child mentioned the need for self-care, associated support, and resources. Healthcare professionals mentioned that during busy shifts, they had to transition between life and death in different rooms, and midwives in our study expressed the desire to dedicate one shift solely to a grieving parent, though structural constraints typically prevented this:

*‘I don’t like to do it [caring for bereaved parents] because I find it emotionally quite burdensome. I would like to do that if I knew I have my whole shift to deal with this. But there are births in between, there are monitors in between …’* (Professional 5, midwife)

*‘… and my biggest fear is not being able to do something right with such a stillbirth or an in-utero death or whatever, you only have one chance. If I don’t see that those papers are in order, that something is going wrong, or if the Dostinex is forgotten or the photos are deleted or if that little coffin isn’t what the parents actually wanted ... you have so many opportunities to mess it up, which puts a lot of pressure on you.’* (Professional 5, midwife)

Several healthcare professionals indicated that novice colleagues are sometimes thrown into the deep end when it comes to guiding parents who lose a child and that it requires training and support. It was deemed crucial for inexperienced colleagues to be accompanied by an experienced professional during their initial encounters with parental loss. Professionals indicated there was little room for these cases into their education program. Midwives indicated the added value of education and training around stillbirth care, including aspects of self-care into their training to enhance patient care.

*Other themes*

During the interviews, participants mentioned several other topics they considered important in losing a child. However, they were not directly related to communication, so they will not be discussed here.

## DISCUSSION

The primary aim of this study was to explore how verbal and non-verbal communication by healthcare professionals impacts the experiences of bereaved parents during perinatal loss. The findings underscore the critical importance of sensitive, patient-centered communication in these emotionally charged situations.

One of the key themes that emerged was the need for healthcare professionals to navigate time according to the parents’ pace. Parents appreciated when they were not rushed and were given the time to process their emotions and make decisions, particularly after receiving devastating news. This aligns with previous research emphasizing the importance of allowing parents to set the pace in difficult situations, fostering a sense of control and respect during a time of profound vulnerability^17,18^.

Recognition of parenthood, even in the absence of a living child, was another crucial aspect for the parents. Healthcare professionals found this challenging, especially when deciding to congratulate the parents explicitly. However, parents greatly valued their parenthood when it was acknowledged, whether by using the baby’s name or by respectfully handling the baby. This finding highlights the delicate balance healthcare professionals must strike between sensitivity and acknowledgment, consistent with existing literature that stresses the importance of validating the parents’ identity despite their loss^19^.

Both parents and healthcare professionals consistently emphasized clear and honest communication. Parents expressed a strong need for thorough, repeated information delivered empathetically. This reflects the complexity of their emotional and cognitive states during perinatal loss, where clarity and repetition help make informed decisions. The study’s findings resonate with existing research, which suggests that effective communication helps in immediate decision-making and contributes to long-term emotional well-being^11,20,21^.

The study also highlighted the importance of continuity of care and the role of non-verbal communication. Parents valued a consistent presence from healthcare professionals who were emotionally attuned and could offer comfort through small gestures, such as a touch on the arm. This reinforces that non-verbal cues can significantly enhance the therapeutic relationship and provide emotional support^22^.

Healthcare professionals, on the other hand, recognized the emotional toll that providing care in these situations can take on them, emphasizing the need for self-care and support. This aspect of the findings is particularly important as it suggests that the well-being of healthcare providers is integral to the quality of care they can offer. Therefore, supporting healthcare professionals through training, debriefing, and structured self-care opportunities could be a valuable area for further development and research^11,23^.

### Strengths and limitations

This is the first Belgian qualitative study to explore experiences regarding professional–parent communication, specifically in perinatal loss. The grounded theory approach used in this study allowed for the emergence of patterns from data, enhancing credibility. We applied maximum variation purposive sampling from different hospital wards to ensure that diverse perspectives were captured. Multidisciplinary inclusion of professionals offered holistic insights into communication practices and challenges. A resonance group of bereaved parents helped us to ensure sensitivity and relevance to experiences. Several potential limitations of the study should be considered. Recall bias is a potential limitation in the study, as participants reported experiences of perinatal loss from the past five years. The emotional weight of their grief may influence their recollections, leading to selective memory of interactions with healthcare professionals. Despite efforts to mitigate this through member checking, the subjective nature of memory may still affect the accuracy of their accounts.

Furthermore, sampling or selection bias might have been mitigated by our choice of maximum variation purposive sampling. However, relying on recruitment through professional associations and hospital wards might have caused relevant groups not to be adequately represented (e.g. lower socioeconomic status). This can limit the transferability of the findings. Partners were not fully accounted for. Although we aimed to explore non-verbal communication, we relied on stories from the participants. Since the interviews were sometimes conducted online due to COVID-19 safety restrictions, this might have hindered appropriate mimicking of the non-verbal behavior they wanted to show the interviewer. We also tried to mitigate socially desirable answers in interviews with healthcare professionals by stating that audio was anonymized, but this cannot fully be diverted. This might have been better captured in an ethnographic research design observing communication. To truly understand the (verbal) communication, applying in-depth Conversation Analysis (CA) might have been valuable^24^. Finally, this study did not capture macro-level differences in healthcare systems and societal norms that can influence communication practices and experiences surrounding death and dying^25^.

### Implications for clinical practice and future research

Ongoing training for healthcare professionals is crucial to enhance the support provided to bereaved parents. In professional development programs, we recommend incorporating specific content focused on effective verbal and non-verbal communication strategies related to perinatal loss. Our institution offers workshops that aim to equip healthcare providers, such as midwives, with the necessary skills to navigate these sensitive conversations with compassion and empathy (see also the Supplementary file for support offered in Belgium).

Furthermore, future research should investigate the impact of sociodemographic factors on the experiences and support needs of bereaved parents. It is also essential to study partners as a distinct group to better understand their unique challenges and perspectives in the context of perinatal loss. This research could inform more tailored and inclusive approaches to care.

## CONCLUSIONS

This study underscores the vital role of compassionate, patient-centered care in supporting bereaved parents after perinatal loss. Clear, honest, and sensitive communication is crucial, as parents value understandable information that facilitates informed decision-making. Recognizing parenthood, using the baby’s name, and showing respect significantly aided the grieving process. Both verbal and non-verbal communication played pivotal roles. Parents appreciated empathetic healthcare professionals, who allowed them time to process their emotions and offered small gestures of comfort and authenticity. These findings underscore the importance of ongoing training for healthcare professionals in handling sensitive situations. Our study highlights the significance of empathetic communication and offers insights for enhancing professional-parent interactions in perinatal loss guiding improvements in clinical practices and training programs to ensure comprehensive support.

## Supplementary Material



## Data Availability

The data supporting this research are available from the authors on reasonable request.
