# Supplementary material for: A qualitative study of bereaved parents and healthcare professionals on perinatal loss
Source: Eur J Midwifery. 2024 Dec 19;8:10.18332/ejm/194159. doi: 10.18332/ejm/194159 (PMC11656490; doi:10.18332/ejm/194159)
Supplement: Supplementary file 1 [file EJM-8-76-s1.pdf]

ADDENDUM

Bereavement care plans for parents who lost a child

Bereavement care plans for parents in Belgium are an essential component of the support system available to those who have experienced the devastating loss of a child. These plans provide comprehensive support, addressing the emotional, psychological, practical, and legal challenges faced by grieving parents. Backed by a strong legal framework and supported by community organizations and societal awareness, bereavement care in Belgium aims to help parents navigate their grief.

Legal Framework Supporting Bereavement Care for Parents

In Belgium, the legal framework plays a significant role in shaping the support available to parents who have lost a child. There are several key aspects of Belgian law that directly impact bereavement care for parents.

1. Parental Bereavement Leave: One of the most important legal provisions in Belgium is the entitlement to parental bereavement leave. Belgian law allows parents to take time off work following the death of a child, providing them with the space they need to grieve without the added pressure of returning to work immediately. The length of bereavement leave can vary depending on the circumstances, but it is generally recognized that parents require a longer period of time compared to other types of bereavement. During this leave, parents are entitled to continue receiving their salary, ensuring that financial concerns do not compound their grief.

2. Financial Support and Benefits: In addition to bereavement leave, the Belgian government provides various forms of financial support to parents who have lost a child. This can include assistance with funeral costs, which can be a significant burden for grieving families. Parents may also be eligible for certain social security benefits, particularly if they were dependent on the child for financial support. These benefits are designed to alleviate some of the practical challenges that arise after a loss, allowing parents to focus on their emotional healing.

3. Legal Rights Regarding Funeral Arrangements: Belgian law provides clear guidelines on the rights of parents when it comes to making funeral arrangements for their child. Parents have the right to decide on all aspects of the funeral, including whether the child will be buried or cremated, the location of the burial, and the nature of the funeral service. The law also ensures that parents are informed and involved in any post-mortem examinations or procedures that may be required, providing them with a sense of control and involvement in their child's final journey.

4. Registration and Recognition of the Loss: In Belgium, the loss of a child, including stillbirths and neonatal deaths, must be officially registered with the civil authorities. This legal recognition is important for parents, as it acknowledges the existence of their child and the significance of their loss. The registration process also triggers the availability of certain legal and financial benefits, ensuring that parents can access the support they are entitled to.

Societal and Community Support

In addition to the legal framework, societal and community support play a vital role in the bereavement care available to parents in Belgium. Belgian society, with its strong emphasis on family and community, provides a supportive environment for grieving parents.

1. Community Organizations and Support Groups: Belgium has a number of community organizations and support groups specifically dedicated to helping parents who have lost a child. These organizations offer a range of services, including counseling, support groups, and practical assistance. They also provide a space for parents to connect with others who have experienced similar losses, offering mutual support and understanding. Examples of such organizations include "Parents d'Enfants Décédés" (Parents of Deceased Children) and "Berrefonds” who support bereaved parents in Belgium.

2. Religious and Spiritual Support: Given Belgium’s diverse religious landscape, spiritual support is an important aspect of bereavement care for many parents. Religious institutions often provide comfort and guidance to grieving families, offering services such as memorial masses, prayers, and spiritual counseling. For parents who are not religious, alternative forms of spiritual support, such as mindfulness or meditation practices, may be available through community organizations or specialized counselors.

3. Public Awareness and Education: Public awareness and education about the impact of child loss are crucial in creating a supportive societal environment for grieving parents. In Belgium, there has been increasing recognition of the need to educate the public about the challenges faced by bereaved parents and to promote a culture of empathy and support. Campaigns and initiatives aimed at raising awareness about child loss and its impact are helping to reduce the stigma associated with grief and encouraging more open conversations about this difficult subject.

© 2024 Jansens F. et al.
